# Role of Antimicrobial Peptides in Skin Barrier Repair in Individuals with Atopic Dermatitis

**DOI:** 10.3390/ijms21207607

**Published:** 2020-10-14

**Authors:** Hai Le Thanh Nguyen, Juan Valentin Trujillo-Paez, Yoshie Umehara, Hainan Yue, Ge Peng, Chanisa Kiatsurayanon, Panjit Chieosilapatham, Pu Song, Ko Okumura, Hideoki Ogawa, Shigaku Ikeda, François Niyonsaba

**Affiliations:** 1 Atopy (Allergy) Research Center, Juntendo University Graduate School of Medicine, Tokyo 113-8421, Japan; haidalieuhue0710@gmail.com (H.L.T.N.); taneiro87@hotmail.com (J.V.T.-P.); y-umeha@juntendo.ac.jp (Y.U.); yhn125300@163.com (H.Y.); g-peng@juntendo.ac.jp (G.P.); kokumura@juntendo.ac.jp (K.O.); ogawa@juntendo.ac.jp (H.O.); ikeda@juntendo.ac.jp (S.I.); 2 Department of Dermatology and Allergology, Juntendo University Graduate School of Medicine, Tokyo 113-8421, Japan; 3 Institute of Dermatology, Department of Medical Services, Ministry of Public Health, Bangkok 10400, Thailand; chanisa.kiatsurayanon@gmail.com; 4 Department of Microbiology, Faculty of Medicine, Chiang Mai University, Chiang Mai 50200, Thailand; chillipop4507@gmail.com; 5 Department of Dermatology, Xijing Hospital, Fourth Military Medical University, Xi’an 710032, China; songpu@fmmu.edu.cn; 6 Faculty of International Liberal Arts, Juntendo University, Tokyo 113-8421, Japan

**Keywords:** antimicrobial peptide, atopic dermatitis, barrier function, epidermal barrier, filaggrin, skin barrier repair

## Abstract

Atopic dermatitis (AD) is a common chronic inflammatory skin disease that exhibits a complex interplay of skin barrier disruption and immune dysregulation. Patients with AD are susceptible to cutaneous infections that may progress to complications, including staphylococcal septicemia. Although most studies have focused on filaggrin mutations, the physical barrier and antimicrobial barrier also play critical roles in the pathogenesis of AD. Within the physical barrier, the stratum corneum and tight junctions play the most important roles. The tight junction barrier is involved in the pathogenesis of AD, as structural and functional defects in tight junctions not only disrupt the physical barrier but also contribute to immunological impairments. Furthermore, antimicrobial peptides, such as LL-37, human β-defensins, and S100A7, improve tight junction barrier function. Recent studies elucidating the pathogenesis of AD have led to the development of barrier repair therapy for skin barrier defects in patients with this disease. This review analyzes the association between skin barrier disruption in patients with AD and antimicrobial peptides to determine the effect of these peptides on skin barrier repair and to consider employing antimicrobial peptides in barrier repair strategies as an additional approach for AD management.

## 1. Introduction

Atopic dermatitis (AD) is a highly prevalent, chronic inflammatory skin disease characterized by the complex interplay between skin barrier disruption and immune dysregulation [1,2,3]. From the first report in 2006, describing a loss-of-function mutation in the gene encoding the filament aggregating protein filaggrin (FLG), the role of FLG in the barrier-based pathogenesis of AD has been extensively researched [2,4]. Within the epidermis of human skin, FLG is an indispensable component of the epidermal differentiation complex. FLG generates natural moisturizing factor (NMF) and plays a critical role in epidermal barrier function [5]. Furthermore, FLG mutations increase the risk of early initiation of AD, which may increase the severity and persistence of the disease [4,6,7,8]. In addition to the research characterizing FLG, the physical barrier and antimicrobial barrier have also been determined to play crucial roles in the barrier-based pathogenesis of AD. Regarding the physical barrier, the stratum corneum (SC) and tight junctions (TJs) have been reported to play the most important role [9]. The TJ barrier has consistently been shown to be involved in the pathogenesis of AD, in which the structural and functional defects in TJs not only disrupt the physical barrier but also contribute to immunological impairments [10,11,12].

Antimicrobial peptides (AMPs) are considered a rapid and first-line response of the innate immune system to microbial pathogens. Together with their antimicrobial effects, AMPs also exert immunomodulatory effects by inducing cell migration, proliferation, and differentiation, regulating cytokine/chemokine production, improving angiogenesis and wound healing, and sustaining the barrier function of the skin [13,14]. Interestingly, various studies have demonstrated that AMPs, such as cathelicidin LL-37, human β-defensin (hBD)-1, hBD-3, and S100A7 protein, increase the levels of TJ-related proteins and promote epidermal barrier function [15,16,17,18]. In addition, although many advances in the understanding of skin barrier dysfunction in the pathogenesis of AD have been achieved, the treatment of this chronic disease remains unsatisfactory. In this review, we highlight the association between skin barrier dysfunction in patients with AD and skin-derived AMPs to consider employing these peptides in barrier repair strategies as an additional therapeutic approach for AD.

## 2. Skin Barrier

**Figure 1:**
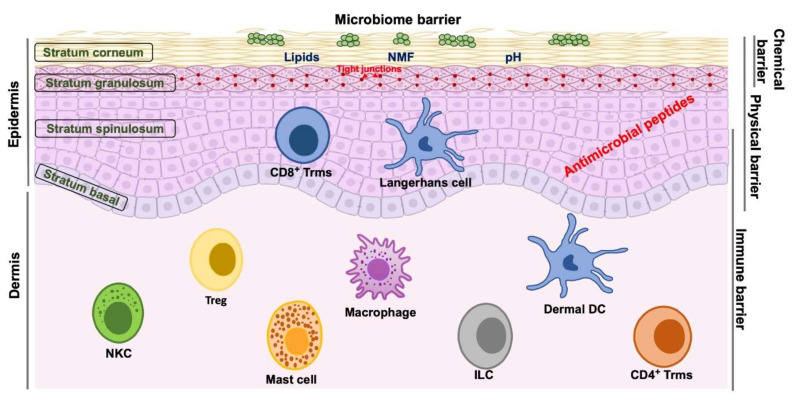
Components of the cutaneous barrier. DC: dendritic cell, ILC: innate lymphoid cell, NKC: natural killer cell, NMF: natural moisturizing factor; Treg: regulatory T-cell; Trm: resident-memory T-cell.

Human skin, the largest organ of the human body, constitutes a pivotal barrier against environmental pathogens. Functionally, the cutaneous barrier is divided into four different levels: the microbiome barrier, the chemical barrier, the physical barrier, and the immune barrier (Figure 1). The microbiome barrier is the outermost layer of the skin, consisting of numerous microbial communities, and it functions as the first active defense against environmental invaders [19]. Various reports have described how commensal microbes of the skin microbiome interact with pathogenic bacteria. As shown in the study by Iwase et al., the serine protease Esp secreted by *Staphylococcus epidermidis (S. epidermidis)*, a commensal bacterium, impedes biofilm formation by *Staphylococcus aureus (S. aureus)* [20]. On the other hand, normal bacteria of the skin, such as *S. epidermidis*, also inhibit inflammatory cytokines expressed by human keratinocytes [21].

The chemical barrier consists of AMPs, NMF, factors that contribute to cutaneous pH, and epidermal lipids [9]. Notably, NMF is located within corneocytes, and changes in SC NMF levels alter cutaneous pH and SC lipids. Other constituents of the NMF, such as lactate and potassium, also play important roles in maintaining the hydration and physical features of the SC [9,22,23]. SC lipids primarily include free fatty acids, ceramides, and cholesterol. Corneocytes, which are derived through epidermal differentiation, are surrounded by a cornified envelope that exhibits chemical crosslinking with a monolayer of nonpolar lipids (ω-hydroxylated ceramides and free fatty acids) [24,25,26]. AMPs have been shown to play important roles in both antimicrobial and immunomodulatory activities, as discussed in the next section.

The SC and TJ proteins are essential components of the physical barrier (Figure 1). The SC consists of mature, denucleated, flattened keratinocytes, and their membranes are replaced by a “cornified envelope”, resulting from the epidermal differentiation process [27]. The stratum granulosum (SG), the next layer below the SC, is composed of granules containing FLG, loricrin, and keratin filaments and laminar bodies with lipids, corneodesmosin, and kallikrein. Keratinocytes in the SG primarily produce triglycerides and cholesterols, indicating a crosslink between the physical and chemical barriers. Within the SG, most TJ proteins are transmembrane proteins and connect adjacent keratinocytes. TJ proteins are classified into the claudins, occludin, and zonula occluden families and are thought to form a barrier regulating the transport of water and solutes [28].

The immune barrier is dynamic and comprises several resident cell populations of the cutaneous epidermis and dermis (Figure 1). In addition to recruiting immune cells, keratinocytes also promote their viability and persistence. For example, tissue-resident memory T (Trm) cells are long-lived cells that are produced after the clearance of an infection and remain in the skin to exert protective immune effects. Trm cells not only accumulate in the infected area but are also allocated broadly to healthy skin areas to facilitate protection against secondary microbial contamination [29,30]. Because of the wide distribution of resident immune cells within skin layers, the immune barrier is highly interconnected with other levels of the cutaneous barrier to maintain skin homeostasis. Following disruption of the physical barrier, pathogenic invaders might activate resident immune cells, particularly Langerhans cells (LCs), which subsequently initiate the T-cell response. In addition to mounting this essential response, resident cells of the immune barrier also promote barrier repair and homeostasis [31].

## 3. Skin Barrier Dysfunction in Individuals with AD

In 2006, loss-of-function mutations in the FLG gene were first described as etiological factors of ichthyosis vulgaris, and these mutations were subsequently identified as major risk factors for AD development [4,32]. FLG is a key protein of the epidermal barrier. FLG deficiency is involved in various pathways related to barrier function, including dysregulation of keratinocyte differentiation, impairment of TJ formation, disruption of SC integrity, alterations in lipid formation, induction of a decrease in the level of NMF, and an increase in sensitivity to skin infection [33,34,35,36]. Moreover, the disruption of SC integrity results from a complicated link between an increase in transepidermal water loss (TEWL), skin dehydration, and a decrease in the level of NMF, leading to the xerosis that characterizes AD [37]. Since their first description, FLG null mutations have continued to constitute the strongest genetic risk factor for AD. FLG mutations are presumed to predispose patients to an earlier onset of AD, as well as to prolongation and exacerbation of the disease [38]. However, in addition to FLG mutations, other factors likely contribute to AD development. In this context, a significant number of patients with AD do not carry any of the known FLG mutations, and conversely, approximately 40% of individuals with FLG null mutations do not display any AD manifestations [39].

The TJ barrier is located beneath the SC and forms the specific cell–cell junctions that seal the intercellular space. The arrangement of the TJ proteins reveals the complexity of epidermal barriers. TJs play an important role in the epidermal permeability barrier, functioning as gates for electrolytes, solutes, and hormones [40,41]. Together with their permeability function in the granular layer, these proteins are also involved in various cellular functions. De Benedetto et al. indicated that the silencing of the TJ protein claudin-1 in human keratinocytes enhances cell proliferation [11]. Moreover, the absence of claudin-1 in the lower epidermal layer of the AD-like allergic dermatitis mouse model also significantly altered the levels of the cell differentiation markers keratin 10 and keratin 14 [42]. On the other hand, TJ barrier defects were demonstrated to lead to abnormal formation of the SC by altering polar lipid and profilaggrin processing, suggesting that TJ dysfunction is related to an abnormal SC barrier in AD pathogenesis [12,43]. According to Furuse et al., claudin-1 deficiency leads to an increase in TEWL and mortality in mice exhibiting no structural abnormalities but induces a notable decrease in the function of the skin barrier [44]. Claudin-1 expression was found to be significantly decreased in the lesional skin of patients with AD compared with the healthy skin of nonatopic individuals [45]. A decrease in the level of this TJ protein is also associated with an increased risk of infection by herpes simplex virus type 1 (HSV1) in patients with AD [46].

Dysregulation of the structural proteins of the epidermis mentioned above is generally related to a dysfunctional cutaneous barrier that contributes to typical AD manifestations of dry, inflammatory skin and allergic sensitization to antigens. In fact, FLG null mutations are strongly associated with atopic asthma, allergic rhinitis, and food allergy [47,48]. Importantly, FLG mutations predispose patients to asthma in the context of atopic eczema, but most known asthma-related genes are not associated with AD [49]. Interestingly, several studies have investigated the emergence of both AD and food allergies as part of transcutaneous sensitization to food allergens. Various reports have described the association between the early appearance of AD in childhood and the development of food allergies, particularly peanut, egg, and milk allergies [50,51]. Additionally, Brown et al. indicated that FLG mutations are not only a highly significant genetic risk factor for AD but also a genetic factor for peanut allergy [52]. These results suggest that FLG mutations associated with cutaneous barrier dysfunction increase the risk of transcutaneous allergen exposure.

**Figure 2:**
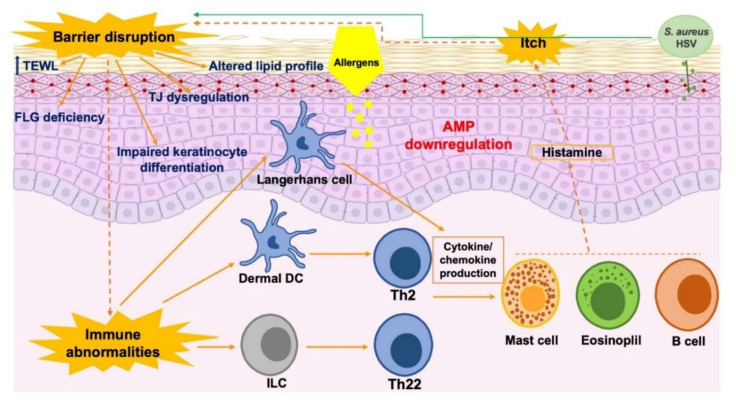
Pathogenic factors of AD and their interactions in skin lesions. AMP: antimicrobial peptide; DC: dendritic cell; FLG: filaggrin; HSV: herpes simplex virus; ILC: innate lymphoid cell; TEWL: transepidermal water loss; Th: T-helper cell; TJ: tight junction.

Taken together, the evidence outlined above indicates that after skin barrier disruption, transcutaneous antigen exposure is facilitated by antigen-presenting cells in the epidermis. Environmental factors subsequently affect skin barrier integrity and may increase both the risk and severity of AD. Among environmental factors, mechanical damage, such as continuous scratching, the use of detergents, and the release of exogenous proteases, are the most notable factors [53]. Moreover, dysfunction of the epidermal barrier might directly elicit cutaneous inflammatory reactions in the setting of AD and activate innate immune responses, including proinflammatory cytokine and chemokine production by keratinocytes and antigen presentation by LCs and dermal dendritic cells (DCs) [54,55,56]. It appears that AD pathogenesis is dominated by CD4^+^ T-helper type 2 (Th2) cells, characterized by the production of interleukin (IL)-4, IL-5, and IL-13 [57,58]. In the context of acute dermatitis, AD skin becomes infiltrated with more Th2 cells and additional CD4^+^ subsets, including Th22 and Th17 cells [57]. Together with CD4^+^ T-cells, other lymphocyte subsets such as type 2 innate lymphoid cells (ILCs) and type 2 cytokine-producing CD8^+^ T-cells were also reported to be increased in AD [59,60]. These cells are thought to be involved in the early sensing tissue damage and initiation of inflammatory reactions prior to the activation of antigen-driven adaptive immune responses. Th2 inflammation in AD also leads to the enrollment of mast cells, eosinophils, and other immune-cell subsets, consequently contributing to the pathological inflammation via the release of mediators such as histamine. Interestingly, proinflammatory cytokines, including IL-31, and histamine may contribute to severe itch in AD [61] (Figure 2). The interplay between skin barrier disruption and immune dysregulation in AD is complicated. Disruption of the epidermal barrier might elicit inflammatory reactions and activate immune responses, and skin inflammation may, in turn, impact skin barrier function. In fact, inflammatory reactions may lead to secondary changes such as suppression of AMP production by Th2 cytokines [62], inhibition of epidermal differentiation complex gene FLG by Th2 and Th22 cytokines [63,64], and induction of epidermal hyperplasia by IL-22 [57], which further provoke AD manifestations.

## 4. Skin Barrier Dysfunction-Related Infections in AD

Skin barrier dysregulation represents a hallmark of AD pathogenesis, in which a deficiency of the AMP and TJ barriers plays a crucial role. Several recent studies have determined the pivotal role of AMPs in the permeability barrier, suggesting a dynamic interaction between the physical barrier and the chemical fence supported by AMPs to protect against infections. In fact, the AMP barrier and permeability barrier functions are both coregulated and independent [65,66]. Colonization by *S. aureus* is a characteristic feature of AD and is observed in both the lesional and nonlesional skin of patients with AD [67]. *S. aureus* colonization can contribute to pruritus and persistent inflammation [68] and may lead to secondary infections in the skin, such as impetiginization, folliculitis, cutaneous abscesses, and cellulitis [69]. Secondary infections in patients with AD subsequently elicit a vicious itch–scratch cycle that may create more entry points for allergens and pathogens, and this cycle further compromises the permeability barrier [68,70]. Colonization with *S. aureus* was indicated to progress to severe infections, including bacteremia, sepsis, endocarditis, arthritis, pneumonia, and ocular infections [71,72]. Similarly, Hoeger et al. reported two cases of *S. aureus* bacteremia in AD patients and suggested that chronic scratching of dry skin in AD may lead to the intrusion of *S. aureus* into the blood circulatory system [72]. Among AD patients with hospital-acquired infections, *S. aureus* bloodstream infections were reported in 60% of cases; the main foci and entry points were skin/soft tissue infections and exclusive intravascular catheters, respectively [73]. Moreover, in 2018, two epidemiological reviews concluded that patients with AD have an increased risk of bacteremia, but the studies did not specify pathogenic factors or clinical manifestations [74,75]. Eczema herpeticum, a particularly serious complication of AD that is caused by an infection with HSV, is associated with viremia, fever, malaise, lymphadenopathy, and significant systemic complications, such as keratoconjunctivitis, meningitis, and encephalitis [76]. Interestingly, Bin et al. found that staphylococcal toxins may promote cutaneous viral replication, suggesting that *S. aureus* colonization might disseminate viral infections in the skin [77]. On the other hand, *Malassezia* spp., a component of the healthy skin flora, was reported to be a trigger factor in AD [78], and sensitization rates against *Malassezia* spp. are particularly higher in patients with head and neck types of AD [79], indicating a correlation between AD and the IgE-mediated sensitization to *Malassezia* spp. [80,81]. Furthermore, prior studies have demonstrated that the impaired skin barrier facilitates *Malassezia* spp. cells to interact with various human skin cells and immune cells, such as keratinocytes, DCs, LCs, natural killer cells (NKCs), and fibroblasts, which consequently maintain the inflammation in patients with AD [82]. Taken together, these findings indicate that infections are a major complication of AD, and characterization of these skin barrier dysregulation-related infections in patients with AD is necessary for prevention and treatment.

## 5. Roles of AMPs in AD

Skin-derived AMPs, also called host defense peptides, are effector molecules that contribute to both innate and adaptive immune responses and perform one of the first defense responses to microbial pathogens. As antimicrobial molecules of the innate immune system, AMPs directly kill microorganisms, including bacteria, viruses, fungi, and parasites. Along with these killing activities, AMPs also exhibit immunomodulatory activities, such as inducing cell migration, proliferation, and differentiation, altering cytokine/chemokine expression, mediating improvements in angiogenesis and wound healing, and regulating cutaneous barrier function. In human skin, AMPs are either constitutively or inducibly expressed by resident and infiltrating cells, specifically, keratinocytes, sebocytes, neutrophils, and mast cells [13,14].

Studies conducted in recent decades have reported an association between AMPs and various skin diseases. A disrupted function of AMPs in patients with AD was first reported by Ong et al. [83], who described the decreased expression of human cathelicidin LL-37 and hBD-2 in patients with AD compared with patients with psoriasis. Additionally, the expression of dermcidin, the AMP derived from the sweat gland, is decreased in patients with AD [84]. These findings suggest the presence of a deficiency in AMP secretion in patients with AD. On the other hand, Gambichler et al. reported a higher level of RNase 7 mRNA in the lesional skin of patients with AD than in patients with psoriasis [85]. Moreover, a study of Kisich et al. [86] reported that constitutive levels of hBD-3 in keratinocytes of both healthy subjects and individuals with AD are similar, and treatment of AD skin using Th2 cytokines inhibited the mobilization of hBD-3 and enhanced the deposition of this AMP onto the surface of *S. aureus*. This study concluded that the high burden of *S. aureus* in patients with AD is caused by the interference of an increase in Th2 cytokines on constitutive killing by keratinocytes. The hampering effect of the Th2 cytokine milieu on AMP expression in AD was also demonstrated by various studies, partially explaining the frequency of infections observed in patients with this disease [62,87,88]. The studies mentioned above suggest that there is not a generalized defect in the expression of AMPs in patients with AD, and the Th2 cytokine milieu may impede the induction of AMPs in AD.

Interestingly, hBD-1, -2, -3, and -4 recruit and activate a broad range of leukocytes. Indeed, hBD-2 induced the chemotaxis of immature DCs, memory T-cells, and Th17 cells through CCR6 [89]. hBD-2 and hBD-3 also recruit myeloid cells, such as monocytes, macrophages, and neutrophils, and function as chemoattractants by binding to CCR2 [90]. Therefore, hBDs contribute to the innate and adaptive immune responses in the skin by functioning as chemoattractants. In addition to hBDs, LL-37 was shown to induce chemotaxis of mast cells through the receptor MrgX2 [91,92]. Notably, hBDs and LL-37 induce the secretion of IL-31 from mast cells, which plays an important role in regulating the itch sensation in patients with cutaneous disorders [93]. Furthermore, LL-37 induces the production of semaphorin 3A, a chemorepulsive factor of the epidermal nerves that is known to be downregulated in AD skin, suggesting a possible itch-suppressing effect of this AMP on patients with AD [94]. Intriguingly, hBDs induce T-cells to produce IL-4, IL-13, and IL-31, which are involved in the pathogenesis of AD [95]. Furthermore, activated T-cells upregulate Th2-related cytokines, including IL-31, IFN-γ, IL-22, and oncostatin M, in the presence of LL-37, implying that this AMP promotes the establishment of an inflammatory cytokine environment in individuals with T-cell-related skin diseases [96]. hBDs and LL-37 also stimulate inflammatory cytokine production in mast cells and improve vascular permeability [93,97]. Taken together, these findings indicate that AMPs are involved in AD pathogenesis, and the overproduction of AMPs may exert negative effects on the inflammatory conditions of patients with AD.

## 6. Influence of AD Treatments on AMPs

The physiopathological mechanisms of AD are very complex, and they are mediated by interactions between genetic factors, skin barrier dysfunction, and immune abnormalities. Many therapeutic options are available for the management of AD, and these treatments are classified into basic skincare, topical therapy, phototherapy, and systemic therapy. However, the selection of an effective and targeted treatment for patients with moderate-to-severe AD remains challenging. The aim of treatments for this disease is to reduce symptoms, prevent exacerbation, control infections, minimize treatment complications, and restore skin barrier integrity. The principles of the therapeutic approach include education, motivated participation of the patient’s families, enhanced skin hydration and barrier function, reduced exacerbation, and treatment of inflammation. Currently, a curative treatment for AD is unavailable. Thus, in clinical practice, distinct recommendations and approaches for the management of the disease are provided to patients.

The primary goal of basic skin therapy is to restore and maintain cutaneous barrier function. Moisturizers containing urea, hyaluronic acids, or ceramides may improve the integrity of the SC [98,99,100]. More recently, various studies have indicated that moisturizer therapy can decrease the severity of flares and reduce the requirement for topical corticosteroids (TCSs) and topical calcineurin inhibitors (TCIs) to treat AD [101,102]. In addition, both TEWL and AMP expression may be modulated by ceramide-dominant emollient application in patients with AD [103]. Therefore, the application of a ceramide-dominant emollient is associated with cutaneous barrier restoration and improvement of the AMP barrier in AD skin and is recommended by several guidelines for AD treatment and prevention [104,105,106].

Until recently, limited treatment options have been available, including TCSs and TCIss, which have been approved worldwide for topical AD therapy. As shown in multiple trials, TCSs may decrease pruritus and improve acute and chronic signs of AD [107,108,109]. However, as AD is a disease requiring long-term treatment, the application of TCSs may cause local cutaneous side effects, such as secondary infection, striae, skin atrophy, telangiectasias, purpura, steroid folliculitis, and acneiform eruptions [110]. Even with short-term use, TCSs were shown to compromise permeability barrier homeostasis and SC integrity. In murine experiments, TCSs may compromise the antimicrobial barrier by downregulating epidermal AMPs, including mouse β-defensin-3 (equivalent to hBD-2) and cathelin-related AMP (CRAMP, equivalent to LL-37) [111,112,113]. TCIs are steroid-sparing, anti-inflammatory agents that are provided as second-line therapy for the acute and chronic phases of AD in individuals who have not responded to other topical therapies or when those therapies are not indicated [114]. Topical tacrolimus has been demonstrated to be safer and more efficacious than low-potency TCSs [115]. In addition to exerting immunomodulatory effects, tacrolimus has also been shown to improve skin barrier function and skin hydration in patients with AD [116]. According to a recent study by Park et al., the expression of hBD-2 is increased after the application of tacrolimus [103]. In addition, pimecrolimus was shown to increase the expression of AMPs, such as LL-37, hBD-2 and hBD-3, in human keratinocytes [117]. Hence, TCI application not only exhibits anti-inflammatory and skin-hydration-restoring effects but is also involved in the normalization of the permeability barrier and the antimicrobial barrier in individuals with AD.

Ultraviolet (UV) phototherapy has been recommended as a treatment option for AD cases that are refractory to basic care and topical therapies [104,118]. Importantly, UVA-1 and narrowband UVB (NB–UVB) are the most effective treatment modalities for improving clinical signs of AD [119,120]. Interestingly, low-dose UVB irradiation was reported to increase AMP expression and improve barrier recovery in the murine epidermis [121]. Additionally, following NB–UVB therapy, the expression of LL-37 and hBD-1 is increased in patients with AD [122,123]. Moreover, Gambichler et al. [122] indicated that NB–UVB phototherapy may normalize the hBD-2 overexpression observed in patients with AD and suggested that the suppression of inflammation and bacterial infections and the recovery of barrier function may be responsible for the normalization of the hBD-2 level. The results of these studies demonstrated that phototherapy using UVB appears to improve the antimicrobial defense barrier in AD skin.

## 7. AMPs in Skin Barrier Repair: An Option for AD Treatment?

As discussed above in Section 4, defects in the AMP barrier clearly play a critical role in AD pathogenesis. Indeed, LL-37, hBD-2, and hBD-3 are downregulated in AD skin lesions compared to psoriasis lesions [83,87,124]. An explanation for the defect of the antimicrobial barrier was the overexpression of Th2 cytokines, such as IL-4, IL-10, and IL-13, which impede the production of AMPs in AD skin [87]. In particular, the reduction in AMP expression may be caused by the topical application of corticosteroids and calcineurin inhibitors, which increase the rate of microbial superinfections observed in individuals with AD [125]. Regarding antimicrobial activities, LL-37 and hBDs exert potent effects on HSV and *S. aureus*, which are common infections occurring in patients with AD [126,127,128,129,130]. Meyer-Hoffert et al. [131] also proposed a possible antimicrobial effect of hBD-3 on molluscum contagiosum virus infection, another infection frequently observed in individuals with AD. Thus, it is likely that the AMP barrier must be restored to prevent frequent infections in patients with AD.

Interestingly, in addition to exerting antimicrobial effects, AMPs such as hBD-1, hBD-3, LL-37, and S100A7 are involved in the mechanism regulating the epidermal barrier by improving TJ barrier function [15,16,17,18]. Since the first report of claudin-1-deficient mice revealed the existence of continuous claudin-based TJs in the epidermis and the important role of these TJs in the barrier function of mammalian skin [44], several studies have supported the indispensability of TJs in cutaneous barrier function [132,133,134,135,136]. In subjects with AD, downregulation of claudin-1, the essential TJ protein of the epidermal barrier, has been observed in the lesional skin of different cohorts [42,45], thereby promoting viral invasion and increasing sensitivity to eczema herpeticum [46]. Notably, by using a mouse model of hapten-induced dermatitis, Yokouchi et al. found that skin inflammation may impair the SC barrier by disrupting the TJ barrier, even in the absence of FLG deficiency [137]. Taken together, these findings indicate that TJ dysregulation plays a critical role in the pathological features of AD.

On the other hand, LL-37 may reduce itching in patients with AD by inducing the expression of semaphorin 3A, a chemorepulsive factor in the epidermal nerves that is downregulated in AD [94]. Additionally, Chen et al. discovered that LL-37 inhibited double-stranded RNA (dsRNA)-induced expression of CXCL8, CXCL10, CCL5, and thymic stromal lymphopoietin in keratinocytes. This study documented the probable involvement of LL-37 in the suppression of Th2 skin inflammation induced by viral or self dsRNA released from damaged cells [138].

The deficiency of the skin barrier generally plays a crucial role in the pathogenesis of AD resulting from several factors, including epidermal gene mutations, deficiency of AMPs, TJ dysregulation, and immune abnormalities. These factors may interact with each other and modify cutaneous barrier function. In addition, skin barrier dysregulation is associated with increased risk and severity of AD and atopic sensitization. Therefore, various recent studies have focused on skin barrier repair to identify a possible therapy for AD. For instance, Frankel et al. [102] investigated the short-term effectiveness of a ceramide–hyaluronic acid emollient foam as a treatment for AD and compared it with 1% pimecrolimus cream; both the ceramide–hyaluronic acid emollient foam and pimecrolimus cream exhibited good efficacy and safety in the treatment of AD in children and adults. Furthermore, numerous clinical studies have described the efficacy of employing a ceramide-dominant emollient as monotherapy, even in patients with moderate-to-severe AD [98,139]. These findings suggest that skin barrier repair may be an effective approach for AD treatment.

**Figure 3:**
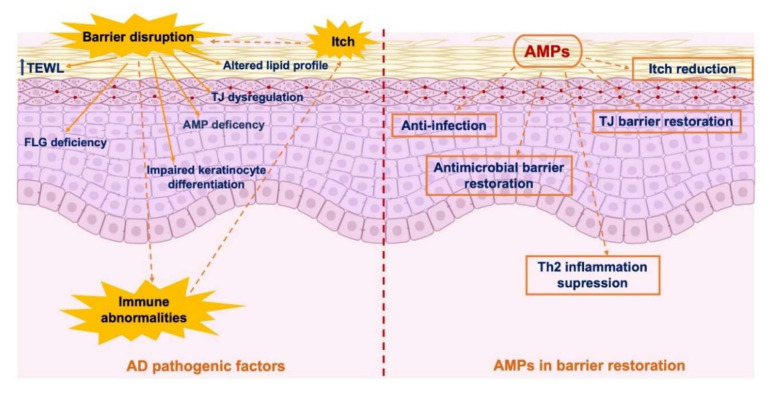
Involvement of antimicrobial peptides in skin barrier restoration in patients with AD. AMP: antimicrobial peptide; FLG: filaggrin; TEWL: transepidermal water loss; Th: T-helper cell; TJ: tight junction.

Taken together, the observations mentioned above indicate that AMPs, including hBDs, LL-37, and S100A7, may restore the skin barrier structure and function and may constitute candidates for barrier repair therapy in AD treatment. Several current guidelines for AD management generally recommend the application of moisturizers as basic mainstay therapy for skin barrier failure, along with anti-inflammatory agents [104,118]. However, regularly used moisturizers were recently reported to compromise the skin if they were applied in a context in which the cutaneous barrier was already injured [140]. Therefore, more potent options for barrier repair therapy in the management of AD must be identified. AMPs not only represent potent antimicrobial agents but are also efficacious at restoring the TJ barrier, reducing itching symptoms, and suppressing Th2 inflammation, and, thus, they may become a new option for barrier repair therapy in AD treatment (Figure 3).

## 8. Conclusions

In recent decades, barrier repair therapy has focused on lipid replacement strategies in patients with AD, with a combination of ceramides, NMF, and pseudoceramide products added to therapeutic moisturizers. However, studies investigating the application of these products in patients with AD have demonstrated their inconsistent efficacy. As AD is a complex disease, skin barrier disruption and immune dysregulation represent the characteristic features of AD, and the treatment of this disease remains challenging. Hence, the ideal and potent skin barrier therapeutic agent currently remains to be investigated. Various observations have suggested the possible option of using AMPs as an additional approach for AD management. In fact, due to their potent antimicrobial activities, AMPs additionally restored the TJ barrier, reduced itching, and suppressed Th2-mediated inflammation. Therefore, further studies are warranted to clarify the involvement of AMPs in the pathogenesis of AD and determine the effect of these peptides on skin barrier repair to determine whether treatment with AMPs constitutes an effective strategy for AD management.

## Abbreviations

AD	atopic dermatitis
AMP	antimicrobial peptide
DC	dendritic cell
dsRNA	double-stranded RNA
hBD	human b-defensin
HDPs	host defense peptides
FDA	US Food and Drug Administration
FLG	filaggrin
HSV	*Herpes simplex virus*
IL	interleukin
LC	Langerhans cell
NKC	natural killer cell
NMF	natural moisturizing factor
*S. aureus*	*Staphylococcus aureus*
SC	stratum corneum
*S. epidermidis*	*Staphylococcus epidermidis*
SG	stratum granulosum
TCI	topical calcineurin inhibitor
TCS	topical corticosteroids
TEWL	transepidermal water loss
Th	helper T-cell
TJ	tight junction
UV	ultraviolet
